# The effect of multi-disciplinary treatment on surgical outcomes and kinematic gait patterns in patients undergoing primary unilateral knee unicompartmental arthroplasty: a retrospective cohort study

**DOI:** 10.3389/fnut.2026.1801572

**Published:** 2026-05-25

**Authors:** Tianxiang Yang, Yulian Ma, Hui Sun, Yinbin Wang, Wentao Gong, Xueqi Liu, Tao Ma, Desheng Chen

**Affiliations:** 1 People’s Hospital of Ningxia Hui Autonomous Region, Third Clinical Medical College of Ningxia Medical University, Yinchuan, China; 2 Department of Orthopedics, People’s Hospital of Ningxia Hui Autonomous Region, Ningxia Medical University, Yinchuan, China; 3 Department of Gastrointestinal Surgery, People’s Hospital of Ningxia Hui Autonomous Region, Ningxia Medical University, Yinchuan, China; 4 Department of Anesthesiology, People’s Hospital of Ningxia Hui Autonomous Region, Ningxia Medical University, Yinchuan, China

**Keywords:** gait, knee osteoarthritis, multi-disciplinary treatment, nutrition, unicompartmental knee arthroplasty

## Abstract

**Objective:**

To investigate the impact of the multi-disciplinary treatment (MDT) approach on surgical outcomes and kinematic gait parameters in patients undergoing primary unilateral unicompartmental knee arthroplasty (UKA).

**Methods:**

Clinical data of patients who underwent UKA at the People’s Hospital of Ningxia Hui Autonomous Region between January 2025 and October 2025 were retrospectively collected and analyzed. Based on whether patients received perioperative MDT management, they were divided into an MDT group (*n* = 40) and a conventional care group (*n* = 50). Perioperative nutrition-related indicators (including albumin, ALB), incidence of perioperative complications, total hospital length of stay, Visual Analogue Scale (VAS) pain scores, Hospital for Special Surgery (HSS) knee scores, knee range of motion (ROM), patient satisfaction, and kinematic gait parameters were measured and compared between the two groups.

**Results:**

The conventional care group showed significantly lower ALB levels on postoperative day 1 and day 3, lower maximum hip flexion, maximum knee flexion, maximum ankle dorsiflexion, lower HSS scores, smaller ROM, and lower satisfaction scores compared to the MDT group. Conversely, the conventional care group had a significantly higher incidence of postoperative complications, longer total hospital stay, and higher VAS pain scores than the MDT group. All these differences were statistically significant (all *p* < 0.05).

**Conclusion:**

The application of an MDT model for elderly patients undergoing primary unilateral UKA can shorten the total hospital stay, reduce the incidence of perioperative complications, promote early postoperative recovery, mitigate postoperative gait kinematic abnormalities, and improve surgical efficacy and patient satisfaction.

## 1 Background

Knee osteoarthritis (KOA) is prevalent among the elderly population. As a commonly seen joint disease in clinical orthopedics, it is a chronic degenerative condition associated with high rates of disability and deformity. Patients suffer from persistent knee swelling, pain, and other symptoms, which severely impact their physical and mental health (1, 2). Studies indicate that the prevalence of KOA in China is as high as 18%, with over 80 million people affected annually, including approximately 200,000 cases of advanced KOA (3, 4). With the progressing aging of the population in China, the familial and societal burden caused by KOA is becoming increasingly substantial. For patients who do not respond to conservative treatment and present significant clinical symptoms, unicompartmental knee arthroplasty (UKA) is one of the most important therapeutic options (5). However, elderly patients often experience declined function in multiple organs and frequently have comorbid conditions involving the respiratory, endocrine, cardiovascular, and other systems preoperatively. This population is prone to postoperative clinical changes, susceptible to surgical complications, and faces considerable perioperative risks (6). Therefore, how to improve the safety and efficacy of surgery for elderly patients during the perioperative period remains a major challenge for clinicians. Multi-disciplinary treatment (MDT) is a novel collaborative model involving healthcare professionals from various disciplines and specialties. It refers to a team of medical staff from different fields who develop comprehensive, dynamic, and optimal treatment plans based on the patient’s specific condition and needs, aiming to enhance surgical outcomes and patient satisfaction, and embodying the patient-centered care philosophy (7). Currently, there is limited clinical research on the correlation between MDT and postoperative recovery and gait. Thus, this study aims to elucidate the impact of MDT on patients’ kinematic gait parameters, providing theoretical support for analyzing its effects on postoperative rehabilitation and biomechanical changes in patients.

## 2 Methods

### 2.1 Patients and study design

Clinical data from patients who underwent primary unilateral UKA for KOA at the People’s Hospital of Ningxia Hui Autonomous Region between January and October 2025 were retrospectively collected. The inclusion criteria were as follows: (1) meeting the diagnostic criteria outlined in the Diagnosis and Treatment Guidelines (2021 edition) (8); (2) KOA diagnosis confirmed by imaging examinations; (3) no significant improvement after conservative treatment and willingness to undergo unilateral knee surgery; (4) meeting the indications for UKA; and (5) provision of informed consent by the patient and family members. The exclusion criteria included: (1) severe hematological disorders; (2) abnormal preoperative complete blood count or long-term use of anticoagulant medications; (3) concomitant tuberculosis, malignancy, or other severe systemic diseases; (4) severe knee joint deformity (9); and (5) incomplete clinical examination or laboratory data.

### 2.2 Treatment method

Based on the treatment model, patients were divided into a Conventional Care Group (Conventional group) and a Multi-disciplinary Treatment Group (MDT group). Patients admitted between January and May 2025 received conventional care, while those admitted between June and October 2025 were managed under the MDT model. This sequential allocation design was adopted for reasons of feasibility and ethics. Once the multidisciplinary protocol was integrated into daily ward routines, it would have been infeasible for the same nursing and therapy staff to strictly maintain a separate conventional care pathway for a subset of patients. Moreover, deliberately withholding the coordinated MDT services from a concurrent control group was deemed ethically inappropriate. The before-after design therefore ensured that the control group remained unexposed to the MDT components. All surgeries were performed by the same surgical team led by a senior attending orthopedic surgeon with over 10 years of experience in knee arthroplasty, following standard operative techniques, and no changes were made to surgical protocols or perioperative care standards between the two study periods (10). The perioperative medication protocols were standardized across both groups. All patients received the same thromboprophylaxis regimen consisting of low-molecular-weight heparin administered at prophylactic dosage. Tranexamic acid was administered according to a standardized protocol of 1 gram intravenous infusion intraoperatively and 1 gram applied locally at wound closure. Uniform multimodal analgesia protocols were applied to all patients, including preemptive analgesia with selective COX-2 inhibitors and periarticular local infiltration analgesia. No patients in either group received glucocorticoids or erythropoietin during the perioperative period.

#### 2.2.1 Conventional group

After admission for KOA, patients underwent routine preoperative examinations and tests. An orthopedic surgeon assessed the patient’s condition. Surgery was scheduled once the patient was clinically stable with no obvious surgical contraindications. Postoperative rehabilitation exercises were guided by the orthopedic surgeon. Throughout the hospitalization, any abnormal test results or clinical changes were addressed via consultations, requesting assistance from relevant departments. No formal nutritional screening, dietary consultation, or structured rehabilitation guidance was provided to patients in the conventional care group.

#### 2.2.2 MDT group

A multi-disciplinary treatment (MDT) model was implemented, led by the Department of Orthopedics and involving physicians from the Departments of Nutrition, Anesthesiology, Rehabilitation Medicine, and Cardiology to form an MDT team. After admission and completion of necessary examinations, the MDT team conducted a comprehensive, multi-disciplinary assessment of the patient’s condition to formulate an optimal, individualized treatment plan. The managing orthopedic surgeon monitored the patient’s condition throughout the perioperative period, communicating and coordinating with specialist physicians as needed. (1) Nutritionist: Conducted preoperative nutritional risk screening using the NRS-2002 tool and Subjective Global Assessment (SGA) within 24 h of admission. For all patients scheduled for UKA, a standardized perioperative nutritional support plan was initiated. The plan included: (a) individualized perioperative dietary guidance regarding meal frequency, dietary composition, and preoperative fasting management; (b) daily preoperative supplementation with a complete nutritional formula providing 370 kcal of energy (protein 21 g, fat 10 g, carbohydrates 46 g); (c) preoperative carbohydrate loading with glutamine 10 g administered 2 h before surgery with 50 mL warm water; and (d) postoperative nutritional support based on gastrointestinal function assessment, with emphasis on early resumption of oral intake. The nutritionist reviewed the patient every 24 h during hospitalization and adjusted the nutritional plan as needed. (2) Rehabilitation Physician: Provided preoperative rehabilitation education within 24 h of admission, including instruction on isometric quadriceps contraction, ankle pump exercises, and proper use of walking aids. Postoperatively, the rehabilitation physician conducted daily bedside visits starting on postoperative day 1, supervising active knee flexion-extension exercises, straight leg raises, and ambulation training with a walker. Exercise intensity and progression were individually adjusted based on the patient’s pain tolerance and clinical status. (3) Anesthesiologist: Conducted a preoperative bedside assessment within 24 h of admission to evaluate anesthetic risk and formulate an individualized anesthetic plan. Intraoperatively, the anesthesiologist closely monitored vital signs. For postoperative pain management, ultrasound-guided nerve block was performed, combined with a multimodal analgesic regimen including selective COX-2 inhibitors. A postoperative visit was conducted within 24 h of surgery to assess pain control using the Visual Analogue Scale, manage any anesthesia-related complications, and adjust the analgesic regimen as needed. (4) Cardiologist: For patients with a known history of hypertension or coronary artery disease, a cardiologist provided consultation within 24 h of admission to optimize perioperative medication management, including decisions regarding continuation or withholding of antiplatelet agents and adjustment of antihypertensive therapy. The cardiologist reviewed the patient’s cardiovascular status daily during the first three postoperative days. All team members performed dynamic assessments and communicated through a shared electronic medical record platform to adjust the treatment plan according to the patient’s clinical progress.

### 2.3 Surgical procedure

All patients underwent elective surgery after exclusion of significant contraindications. The SLED fixed-bearing unicompartmental knee prosthesis (LINK, Germany) was used in all cases. No intraoperative blood transfusions were required in either group.

### 2.4 Discharge criteria

Patients were discharged upon meeting the following criteria: absence of severe anemia, poor wound healing, or other postoperative complications; tolerable knee pain; and achievement of at least 90° of knee flexion (11).

### 2.5 Observational metrics

#### 2.5.1 Nutrition-related indicators

Serum total protein (TP), hemoglobin (Hb), and albumin (ALB) levels were measured preoperatively and on postoperative days 1 and 3.

#### 2.5.2 Kinematic gait parameters

Gait data for all patients were acquired and processed using the Qualisys three-dimensional motion capture system (11). Data collection was synchronized with clinical assessments, performed at admission and on postoperative day 3. The acquired motion parameters included maximum flexion angles of the hip and knee joints, as well as the maximum ankle dorsiflexion angle.

#### 2.5.3 Clinical scoring indicators

The Hospital for Special Surgery (HSS) knee score and knee range of motion (ROM) were assessed.

#### 2.5.4 Perioperative complications

The occurrence of electrolyte disturbances, postoperative blood transfusion, postoperative nausea, postoperative delirium, perioperative hypertension, postoperative deep venous thrombosis (DVT), and pulmonary embolism was recorded.

#### 2.5.5 Other indicators

The total hospital length of stay and Visual Analog Scale (VAS) pain scores were recorded for both groups. Patient satisfaction was evaluated using a 5-point Likert scale (0 points: very dissatisfied, 2 points: dissatisfied, 4 points: neutral, 6 points: satisfied, 8 points: very satisfied).

### 2.6 Data processing methods

#### 2.6.1 Data collection and organization

Clinical information for this study was collected via our hospital’s electronic medical record system and ward-based physical examinations.

#### 2.6.2 Data quality control

All data collection was conducted under the guidance and supervision of clinical and medical records department physicians, using a unified methodology. On the day of data collection completion, a dedicated researcher was responsible for verifying the database, promptly supplementing and correcting any entries. VAS and HSS scoring were completed under the guidance and supervision of senior physicians, strictly adhering to each scoring standard to ensure authenticity and validity.

#### 2.6.3 Statistical analysis

Data were analyzed using SPSS (version 24.0). The normality of continuous variables was assessed using the Shapiro–Wilk test. Data with a normal distribution are presented as mean ± standard deviation. Non-normally distributed data are presented as median and interquartile range. Within-group comparisons were conducted using paired t-tests or the Wilcoxon signed-rank test, as appropriate. Categorical data are presented as frequencies and percentages. A two-tailed *p*-value of less than 0.05 was considered statistically significant. To control for potential confounding factors, multivariate linear regression models were constructed for each primary outcome measure and kinematic gait parameter. The MDT intervention was included as the primary independent variable. All models were adjusted for age, sex, body mass index, disease duration, surgical side, preoperative albumin level, and the presence of hypertension, coronary artery disease, and diabetes mellitus. For clinical outcomes with corresponding preoperative measurements (VAS, HSS, and ROM), the respective preoperative values were additionally included as covariates. For kinematic gait parameters (maximum hip flexion, maximum knee flexion, and maximum ankle dorsiflexion), the corresponding preoperative gait measurements were additionally included. Results are reported as adjusted regression coefficients (*β*) with 95% confidence intervals.

## 3 Results

### 3.1 Patient characteristics

General clinical characteristics, including age, height, weight, and body mass index (BMI), were collected from the patients. No statistically significant differences were observed in the general data between the two groups (all *p* > 0.05) (see Table 1).

**Table 1:** Baseline characteristics of patients.

Characteristic		MDT group (*n* = 40)	Conventional group (*n* = 50)	Statistical value	*p*-value
Age (years)		65.50 ± 6.56	66.58 ± 5.59	*t* = 0.84	0.40
Height (m)		1.60 (1.55, 1.67)	1.61 ± 0.07	*z =* −0.37	0.71
Weight (kg)		63.45 ± 8.17	65.50 (60.00, 70.00)	*z =* −1.64	0.10
BMI (kg/m^2^)		24.52 ± 2.82	25.56 ± 2.97	*t* = 1.69	0.10
Sex [*n* (%)]	Male	9 (22.50)	12 (24.00)	*χ*^2^ = 0.03	0.87
	Female	31 (77.50)	38 (76.00)		
Surgical side [*n* (%)]	Left	22 (55.00)	23 (46.00)	*χ*^2^ = 0.72	0.40
	Right	18 (45.00)	27 (54.00)		
Comorbidities [*n* (%)]	Hypertension	27 (67.50)	30 (60.00)	*χ*^2^ = 0.54	0.46
	Coronary Disease	5 (12.50)	6 (12.00)	*χ*^2^ = 0.01	0.94
	Diabetes	6 (15.00)	7 (14.00)	*χ*^2^ = 0.02	0.89
Disease duration (months)		120.00 (36.00, 144.00)	96.00 (60.00, 120.00)	*z =* −0.11	0.91

### 3.2 Nutrition-related indicators

The between-group comparisons of nutrition-related indicators are shown in Table 2. No statistically significant differences were observed between the two groups in preoperative TP, Hb, or ALB levels (all *p* > 0.05). Similarly, no significant differences were found in TP or Hb levels on postoperative day 1 or day 3 (all *p* > 0.05). However, ALB levels on postoperative day 1 and day 3 were significantly lower in the Conventional group compared to the MDT group (both *p* < 0.05). In the within-group analysis, pairwise comparisons (preoperative vs. postoperative day 1, preoperative vs. postoperative day 3, and postoperative day 1 vs. day 3) of TP, Hb, and ALB levels showed statistically significant differences within each group (all *p* < 0.05).

**Table 2:** Comparison of nutrition-related indicators between the two groups (g/L).

Indicator		MDT group (*n* = 40)	Conventional group (*n* = 50)	Statistical value	*p*-value
TP	Preoperative	65.65(62.09,72.51)	69.01 ± 4.80	*z =* −1.69	0.09
	Postoperative day 1	62.08(58.73,66.73)^*^	60.63 ± 4.82^*^	*z =* −1.64	0.10
	Postoperative day 3	59.53 ± 5.05^*#^	59.35 ± 4.61^*#^	*t* = −0.17	0.87
Hb	Preoperative	134.32 ± 11.59	135.40 ± 15.21	*t* = 0.37	0.71
	Postoperative day 1	124.85 ± 12.27^*^	122.36 ± 14.82^*^	*t* = −0.85	0.40
	Postoperative day 3	104.60 ± 13.85^*#^	103.32 ± 18.07^*#^	*t* = −0.37	0.71
ALB	Preoperative	40.49 ± 4.02	40.28 (38.11, 42.22)	*z =* −0.08	0.94
	Postoperative day 1	36.68 ± 3.67^*^	34.89 ± 3.24^*^	*t* = −2.46	<0.01
	Postoperative day 3	34.46 ± 2.82^*#^	32.88 ± 3.01^*#^	*t* = −2.53	<0.01

^*^Compared with the preoperative value within the same group; ^#^compared with the value on Postoperative Day 1 within the same group.

### 3.3 Kinematic gait parameters

Comparisons of kinematic gait parameters between the groups are summarized in Table 3. No significant differences were observed preoperatively in maximum hip flexion, maximum knee flexion, or maximum ankle dorsiflexion (all *p* > 0.05). Postoperatively, all three parameters were significantly lower in the Conventional group compared to the MDT group (all *p* < 0.05). Within-group comparisons revealed that postoperative values for maximum hip flexion, maximum knee flexion, and maximum ankle dorsiflexion were significantly different from preoperative values in both groups (all *p* < 0.05).

**Table 3:** Comparison of kinematic gait parameters between groups (°).

Parameter		MDT group (*n* = 40)	Conventional group (*n* = 50)	*z*-value	*p*-value
Maximum hip flexion	Preoperative	17.00 (16.00, 23.75)	17.00 (15.75, 22.25)	−0.45	0.65
	Postoperative	25.50 (23.00, 29.75)^*^	22.00 (19.00, 25.00)^*^	−3.96	<0.01
Maximum knee flexion	Preoperative	43.75 ± 5.93	43.00 (35.00, 46.00)	−1.08	0.28
	Postoperative	30.50 (30.00, 33.00)^*^	27.00 (24.00, 30.00)^*^	−4.65	<0.01
Maximum ankle dorsiflexion	Preoperative	15.00 (13.75, 19.00)	15.00 (13.75, 19.00)	−1.78	0.07
	Postoperative	14.00 (11.00, 16.00)^*^	11.00 (9.00, 14.00)^*^	−1.97	<0.05

^∗^Comparison with preoperative.

### 3.4 Clinical scores

Comparisons of clinical scores between the groups are shown in Table 4. No significant differences were found preoperatively in HSS scores or ROM (both *p* > 0.05). Postoperatively, both HSS scores and ROM were significantly lower in the Conventional group than in the MDT group (both *p* < 0.05). Within-group comparisons showed that postoperative HSS scores and ROM were significantly different from preoperative values in both groups (both p < 0.05).

**Table 4:** Comparison of clinical scores between groups.

Indicator		MDT group (*n* = 40)	Conventional group (*n* = 50)	Statistical value	*p*-value
HSS	Preoperative	58.00 (55.00, 64.00)	57.00 (55.00, 63.25)	*z =* −0.60	0.55
	Postoperative	85.00 (83.25, 86.00)^*^	82.00 (78.00, 84.00)^*^	*z =* −4.98	<0.01
ROM (°)	Preoperative	111.63 ± 15.95	109.30 ± 13.40	*t* = −0.75	0.45
	Postoperative	100.00 (95.00, 100.00)^*^	95.00 (90.00,100.00)^*^	*z =* −3.82	<0.01

^∗^Comparison with preoperative.

### 3.5 Perioperative complications

The incidence of postoperative complications was significantly lower in the MDT group compared to the Conventional group (*p* < 0.05). Details are presented in Table 5.

**Table 5:** Comparison of perioperative complications and other indicators between groups.

Indicator		MDT group (*n* = 40)	Conventional Group (*n* = 50)	Statistical value	*p*-value
Complications [*n* (%)]	None	31 (77.50)	24 (48.00)	*χ*^2^ = 7.15	0.03
	One	5 (12.50)	14 (28.00)		
	Two or more	4 (10.00)	12 (24.00)		
Total hospital stay (days)		8.05 ± 1.50	9.00 (8.00, 10.00)	*z =* −2.27	0.02
Satisfaction score		6.00 (6.00, 8.00)	6.00 (4.00, 6.00)	*z =* −4.69	<0.01
VAS	Preoperative	3.00 (3.00, 3.00)	3.00 (3.00, 3.00)	*z =* −0.83	0.41
	Postoperative day 1	3.00 (3.00, 4.00)^*^	4.00 (3.00, 5.00)^*^	*z =* −3.64	<0.01
	Postoperative day 3	4.00 (4.00, 5.00)^*#^	5.00 (4.00, 6.00)^*#^	*z =* −3.54	<0.01

^*^Compared with the preoperative value within the same group; ^#^compared with the value on Postoperative Day 1 within the same group.

### 3.6 Other indicators

As shown in Table 5, the total hospital stay was significantly shorter, and the satisfaction score was significantly higher in the MDT group compared to the Conventional group (both *p* < 0.05). For VAS scores, no between-group difference was observed preoperatively (*p* > 0.05). Postoperatively, VAS scores on both day 1 and day 3 were significantly higher in the Conventional group than in the MDT group (both *p* < 0.05). Within-group comparisons indicated that VAS scores on postoperative day 1 and day 3 were significantly different from preoperative scores, and scores on day 3 were also significantly different from those on day 1 in both groups (all *p* < 0.05).

### 3.7 Multivariate regression analysis

Multivariate linear regression models were constructed to evaluate the independent effect of the MDT intervention on primary outcomes after adjusting for potential confounders. Among clinical outcomes, the MDT intervention remained independently associated with significantly improved values after adjustment. Specifically, MDT was associated with higher postoperative day 1 albumin (adjusted *β* = 1.75 g/L, 95% CI: 0.35 to 3.14, *p* = 0.02) and postoperative day 3 albumin (adjusted *β* = 1.72 g/L, 95% CI: 0.53 to 2.92, *p* = 0.01). MDT was also independently associated with lower pain scores on postoperative day 1 (adjusted *β* = −0.79 points, 95% CI: −1.20 to −0.37, *p* < 0.01) and postoperative day 3 (adjusted β = −0.62 points, 95% CI: −0.96 to −0.28, *p* < 0.01). Functional outcomes at discharge were also significantly better in the MDT group, with higher HSS knee scores (adjusted *β* = 3.38 points, 95% CI: 2.15 to 4.60, *p* < 0.01) and greater knee ROM (adjusted *β* = 4.70 degrees, 95% CI: 2.34 to 7.05, *p* < 0.01). For total hospital length of stay, although the overall multivariate model did not reach statistical significance (model *p* = 0.11), the MDT intervention remained independently associated with a significantly shorter hospital stay (adjusted *β* = −0.77 days, 95% CI: −1.46 to −0.08, *p* = 0.03). Among kinematic gait parameters, the MDT intervention was independently associated with significantly greater maximum hip flexion (adjusted *β* = 4.90 degrees, 95% CI: 2.83 to 6.97, *p* < 0.01) and maximum knee flexion (adjusted *β* = 3.75 degrees, 95% CI: 1.99 to 5.51, *p* < 0.01). The association with maximum ankle dorsiflexion was not statistically significant after adjustment (adjusted *β* = 1.26 degrees, 95% CI: −1.29 to 3.81, *p* = 0.33). Collectively, these multivariate analyses demonstrate that the beneficial effects of the MDT intervention on postoperative nutritional status, pain control, functional recovery, length of stay, and key gait kinematics are largely independent of baseline patient characteristics, comorbid conditions, and preoperative functional status (see Table 6).

**Table 6:** Multivariate regression analysis of MDT intervention on primary clinical and gait outcomes.

Outcome		Adjusted *β* (95% CI)	*p*-value
Clinical outcomes	Postoperative day 1 ALB (g/L)	+1.75 (+0.35, +3.14)	0.02
	Postoperative day 3 ALB (g/L)	+1.72 (+0.53, +2.92)	0.01
	Postoperative day 1 VAS	−0.79 (−1.20, −0.37)	<0.01
	Postoperative day 3 VAS	−0.62 (−0.96, −0.28)	<0.01
	Postoperative HSS	+3.38 (+2.15, +4.60)	<0.01
	Postoperative ROM (°)	+4.70 (+2.34, +7.05)	<0.01
	Total length of stay (days)	−0.77 (−1.46, −0.08)	0.03
Kinematic gait outcomes	Maximum hip flexion (°)	+4.90 (+2.83, +6.97)	<0.01
	Maximum knee flexion (°)	+3.75 (+1.99, +5.51)	<0.01
	Maximum ankle dorsiflexion (°)	+1.26 (−1.29, +3.81)	0.33

All models adjusted for age, sex, body mass index, disease duration, surgical side, preoperative albumin level, hypertension, coronary artery disease, and diabetes mellitus. For clinical outcomes (VAS, HSS, ROM), the corresponding preoperative value was additionally adjusted. For gait outcomes, the corresponding preoperative gait value was additionally adjusted.

## 4 Discussion

Knee osteoarthritis (KOA) is a chronic degenerative joint disease characterized by articular cartilage lesions and osteophyte formation. It is prevalent among the elderly, and persistent knee pain and functional decline severely compromise patients’ quality of life and health (2, 12, 13). Surgical intervention is often employed for patients with severe symptoms and significant joint dysfunction. Elderly patients typically exhibit declined physiological function across multiple organ systems, manifesting as reduced resistance and suboptimal nutritional status. For these patients, who frequently have complex multisystem comorbidities, the traditional treatment model often lacks a holistic and comprehensive approach to individual care. This can lead to delays in diagnosis and treatment when symptoms are subtle or clinical changes are not overt, potentially resulting in prolonged treatment courses, suboptimal rehabilitation, increased perioperative complications, and ultimately diminished surgical efficacy and patient satisfaction (7, 14). Consequently, the Multi-disciplinary treatment (MDT) approach has emerged as a key trend towards refined perioperative management and enhanced recovery for KOA patients.

Albumin is the most abundant circulating protein in the body, constituting approximately half of the total plasma protein. It serves as a primary indicator of nutritional status and possesses physiological properties including maintaining colloid osmotic pressure, anti-inflammatory, antioxidant, and anti-platelet aggregation effects (15, 16). Hypoalbuminemia is diagnostically defined as an albumin (ALB) level below 35 g/L (17). In this study, calculation of the mean postoperative ALB levels revealed that the Conventional group’s means on both postoperative day 1 and day 3 were below 35 g/L. In contrast, only the postoperative day 3 mean in the MDT group fell below this threshold. The statistically significant differences in postoperative ALB levels between the groups suggest that patients in the MDT group experienced less postoperative decline in this key nutritional marker. Rather than reflecting the isolated effect of nutritional supplementation alone, this finding likely represents the combined benefits of improved perioperative pain control, earlier mobilization under the guidance of the rehabilitation physician, reduced surgical stress response, and targeted nutritional support afforded by the coordinated, multi-specialty MDT model. The pain and functional limitations associated with KOA often lead to significant gait alterations. Patients typically reduce knee flexion and increase knee extension during gait to alleviate pain and enhance joint stability, ultimately shifting load-bearing to the contralateral limb. In this study, while maximum hip flexion angle, VAS scores, and KSS scores showed postoperative improvement compared to preoperative values, maximum knee flexion angle, maximum ankle dorsiflexion angle, and ROM decreased postoperatively. This may be attributable to the inherent surgical trauma of UKA in elderly patients and the extended time required for postoperative rehabilitation. Research indicates that functional recovery after UKA continues beyond 6 months up to 2 years, with knee flexion range typically peaking around 1 year postoperatively (18). However, compared to the Conventional group, the MDT group demonstrated more pronounced improvement in activity-related VAS scores and clinical scores, alongside a lesser degree of gait parameter abnormality. The MDT model for UKA patients provides extensive perioperative health guidance and promotes more active postoperative knee exercise, facilitating the recovery of overall physiological function. This leads to significant pain relief, improved subjective well-being, a reduction in pain-induced protective mechanisms and compensatory clinical presentations, and consequently, better postoperative gait parameters and clinical scores—findings consistent with our previous research (11). Postoperative complications frequently impact surgical outcomes and patient satisfaction, with severe complications potentially compromising surgical safety. Therefore, minimizing complications is a universal goal in surgery and a key strategy for improving outcomes and satisfaction. This study found a lower incidence of postoperative complications and higher pre-discharge HSS and satisfaction scores in the MDT group compared to the Conventional group, indicating that the MDT approach can reduce perioperative complication rates and enhance both surgical efficacy and patient satisfaction.

The MDT model is particularly suitable for elderly perioperative patients who are of advanced age, have multiple comorbidities, and present with complex conditions. It helps overcome the limitations inherent to any single specialty’s perspective. Building upon the expertise of individual specialties, the MDT provides integrated, comprehensive care centered on the patient—a “multiple-to-one” collaborative model. This interdisciplinary cooperation elevates the overall standard of care, leading to better and more efficient healthcare delivery (19–21). By applying the MDT model to UKA patients, this study pooled expertise from orthopedics, anesthesiology, rehabilitation, and other disciplines to formulate holistic and comprehensive treatment plans. This approach resulted in reduced perioperative complications, shorter hospital stays, improved postoperative gait abnormalities, and enhanced surgical outcomes and patient satisfaction.

The multivariate regression analyses performed in this study provide further support for the independent benefit of the MDT intervention. After adjusting for baseline demographic and clinical characteristics, including preoperative functional status, the MDT approach remained significantly associated with improved nutritional markers, reduced pain, better functional scores, greater range of motion, and shorter hospital stay. Importantly, the kinematic advantages observed in the univariate analysis for maximum hip and knee flexion also persisted after multivariate adjustment, underscoring the robustness of the MDT effect on early gait recovery. The lack of a statistically significant independent association with maximum ankle dorsiflexion may reflect the greater influence of acute postoperative factors such as swelling and pain on ankle kinematics, or it may be attributable to limited statistical power. Overall, the consistency of findings across multiple outcome domains reinforces the conclusion that the coordinated, multi-disciplinary care model independently contributes to enhanced early recovery in this elderly surgical population. Although the between-group differences in early kinematic parameters such as maximum knee flexion (approximately 3–4 degrees) were statistically significant, whether these modest early improvements translate into clinically meaningful long-term functional gains requires further investigation with extended follow-up.

It is also important to note that this study did not evaluate the potential influence of patellar height on postoperative functional recovery. Changes in patellar height following knee arthroplasty have been shown to affect clinical outcomes. A recent study demonstrated that different radiological indices of patellar height can predict diverse patient outcomes following knee arthroplasty, with a decreased modified Blackburne-Peel ratio being associated with poorer functional scores and a higher risk of patient dissatisfaction (22). Similarly, research has shown that a decrease in patellar height following UKA may be associated with reduced stair climbing scores at mid-term follow-up (23). Given that our study focused primarily on kinematic parameters of the hip, knee, and ankle joints without specific radiographic assessment of patellar height, the potential confounding effects of patellar height changes on the observed outcomes cannot be excluded. Future studies should incorporate patellar height as a key radiographic assessment parameter and include it in multivariate analyses to better elucidate its independent contribution to postoperative functional recovery and gait biomechanics.

Perioperative medication management represents a critical component of Enhanced Recovery After Surgery protocols and may significantly influence postoperative outcomes. In the present study, the perioperative medication regimens were fully standardized across both groups, with no significant differences in thromboprophylaxis, tranexamic acid administration, or analgesia protocols. The observed improvements in the MDT group are attributable to the comprehensive multi-disciplinary care model itself rather than to differences in pharmacological management. Nevertheless, the potential confounding effects of perioperative medications should be carefully considered in future prospective studies, and detailed documentation of medication regimens should be incorporated into multivariate analyses when group differences exist.

This study has several limitations. First, the assessment metrics were not exhaustive, as other relevant gait parameters were not included. Second, the follow-up duration was relatively short, and longer-term effects require further observation. Third, patients were allocated to treatment groups based on their admission period. This sequential allocation design may introduce temporal bias. Although all surgeries were performed by the same surgical team led by a senior attending orthopedic surgeon with over 10 years of experience in knee arthroplasty, and no changes were made to surgical protocols or perioperative care standards between the two study periods, the potential influence of unmeasured temporal trends on the observed outcomes cannot be completely excluded. Finally, the sample size was modest, and the single-center design may introduce inherent statistical bias, limiting the generalizability of the findings for broader clinical guidance. Future research should involve large-scale, multi-center studies to enhance the robustness and external validity of the results. Expanding the scope of assessment to include a wider range of functional and biomechanical parameters would allow for a more in-depth understanding of the correlations among these factors.

## 5 Conclusion

For elderly patients undergoing primary unilateral UKA, the application of an MDT model can shorten the total hospital stay, reduce the incidence of perioperative complications, promote early postoperative recovery, mitigate early postoperative gait kinematic abnormalities, and improve surgical efficacy and patient satisfaction. This approach aligns with the principles of Enhanced Recovery After Surgery and is worthy of broader clinical application.

## Data Availability

The raw data supporting the conclusions of this article will be made available by the authors, without undue reservation.
